# Body composition analysis in head and neck squamous cell carcinoma

**DOI:** 10.1007/s00405-013-2815-3

**Published:** 2013-11-22

**Authors:** Teresa Malecka-Massalska, Agata Smolen, Kamal Morshed

**Affiliations:** 1 Physiology Department, Medical University of Lublin, Radziwiłłowska 11 Str., 20-080 Lublin, Poland; 2 Department of Mathematics and Biostatistics, Medical University of Lublin, Lublin, Poland; 3 Otolaryngology Department, Head and Neck Oncology, Medical University of Lublin, Lublin, Poland

**Keywords:** Bioelectrical impedance analysis, Impedance vector, Body composition analysis, Head and neck cancer, Squamous cell carcinoma

## Abstract

Direct bioimpedance measures [resistance, reactance, phase angle] determined by bioelectrical impedance analysis (BIA) detect changes in tissue electrical properties. Bioelectrical impedance analysis vector (BIVA) technique is a promising tool, using the pure data obtained by BIA evaluation for the screening and monitoring of nutrition and hydration status. BIVA has the potential to be used as a routine method in the clinical setting for the assessment and management of body fluids. The study was conducted to evaluate soft tissue hydration and mass through pattern analysis of vector plots as height, normalized resistance, and reactance measurements by bioelectric impedance vector analysis in patients with head and neck cancer. Whole body measurements were made with ImpediMed bioimpedance analysis in 134 adult, white, male subjects 22–87 years old: 67 patients with head and neck cancer (H&NC) and 67 healthy volunteers matched by sex, age and BMI as a control group. All patients were previously untreated and without active nutritional interventions. Mean vectors of H&NC group versus the control group were characterized by an increased normalized resistance component with a reduced reactance component (separate 95 % confidence limits, *P* < 0.05). BIVA may offer objective measures to improve clinical decision-making and predict outcomes. In patients with H&NC to reduce post-operational complications monitoring bioimpedance vector trajectory may support therapy planning of individual patients before surgery.

## Introduction

Worldwide, an estimated 6,44,000 new cases of head and neck cancer (H&NC) are diagnosed each year, with two-thirds of these cases occurring in developing countries [1]. In the US, H&NC accounts for 3.2 % (39,750) of all new cancers and 2.2 % (12,460) of all cancer deaths [2]. Malnutrition is common in patients with H&NC. Nutritional deficits have a significant impact on mortality, morbidity, and quality of life in patients with H&NC [3].

Methods to measure and monitor nutritional status can play an important role in the recovery and quality of life for this patient population. Bioelectrical impedance analysis (BIA) has been established as a valuable tool in the evaluation of body composition and nutritional status in many patients’ conditions including cancer of gastrointestinal tract, lung, cancer of pleura and ureter [4–6]. BIA evaluates body components such as resistance (*R*) and reactance (*X*_c_) by recording a voltage drop in applied current [7]. Resistance is the opposition to the flow of an electric current, primarily related to the amount of water present in the tissues. Reactance is the resistive effect produced by the tissue interfaces and cell membranes [8]. Reactance causes the current to lag behind the voltage creating a phase shift, which is quantified geometrically as the angular transformation of the ratio of reactance to resistance, or phase angle (PA).

Bioelectrical impedance vector analysis (BIVA) technique is a promising tool, using the pure data obtained by BIA evaluation for the screening and monitoring of nutrition and hydration status. BIVA has the potential to be used as a routine method in the clinical setting for assessment and management of body fluids [9]. Bioelectrical impedance vector analysis allows non-invasive evaluation of soft tissue hydration and mass through pattern analysis of vector plots as height, normalized resistance, and reactance measurements [10]. BIVA has been used to allow detection, monitoring, and control of hydration and nutrition status using vector displacement for the feedback on treatment in peritoneal dialysis patients [11] and in cancer patients [12].

In particular, phase angle measured at 50 kHz, because of its reproducibility quality, has been used to determine and predict both the state of health in a healthy population and an altered state observed in the diseased population, with diseased conditions including cancer [10–12].

The aim of our observational study was to perform bioelectrical impedance analysis to investigate whether the position on the resistance–reactance (*R*–*X*_c_) plane of impedance vectors from adult male patients with H&NC differed from healthy male age- and body mass index (BMI)-matched control subjects.

## Patients and methods

### Study design

This observational study investigated whether the position on the *R*–*X*_c_ plane of impedance vectors from adult male patients with H&NC differed from healthy male age- and BMI-matched control subjects. No interventions were made based on the impedance data of patients.

### Study populations

Between October 2009 and September 2012, 134 subjects underwent examination of tissue electrical properties. Sixty-seven pre-surgical male patients with H&NC were examined between the age 37 and 74. The histological diagnosis of these patients was squamous cell carcinoma (SCC). This study included 28 patients with laryngeal SCC, 21 patients with oropharyngeal SCC, and 18 patients with oral cavity SCC. The type and location of the cancer, the stage of the disease, and the cancer’s grade are the factors that can influence the results.

All patients were treated at the Otolaryngology Department, Head and Neck Oncology, of the Medical University of Lublin.

Sixty-seven healthy male subjects from the same region matched by age and BMI were selected as the control group for this study. The group of patients with H&NC underwent a baseline nutritional assessment, which included laboratory measurements of serum albumin, transferrin and total protein, subjective global assessment (SGA), and BIA. None of the patients received any nutritional support during the pre and post-operative period. The control group underwent a baseline nutritional assessment, which included SGA and BIA.

This study was conducted according to the guidelines set forth in the declaration of Helsinki, and all procedures involving human subjects/patients were approved by the Research Ethics Committee of the Medical University of Lublin, Poland. All patients gave their written informed consent as a precondition of participation in the study.

### Bioimpedance

Bioelectrical impedance analysis was performed by a medical doctor using ImpediMed bioimpedance analysis SFB7 BioImp v1.55 (Pinkenba Qld 4008, Australia). BIA was performed after a 10-min rest period while the patients were lying supine on a bed, with their legs apart and their arms not touching their torso. All evaluations were conducted on the patients’ right side using the four surface standard electrode (tetra polar) technique on the hand and foot. *R* and *X*_c_ were measured directly in ohms at 5, 50, 100, 200 kHz. *R* and *X*_c_ values were measured three times in each patient, and the mean values were used. PA was obtained from the arc–tangent ratio *X*_c_:*R*. To transform the result from radians to degrees, the result that was obtained was multiplied by 180°/*π*.

### Bioelectrical impedance vector analysis

According to the *RX*_c_ graph method [21], measurements of *R* and *X*_c_ were standardized by the *H* subjects (i.e., *R*/*H* and *X*_c_/*H*) and expressed in ohms per meter. By using the bivariate normal distribution of *R*/*H* and *X*_c_/*H*, we calculated the bivariate 95 % confidence limits for mean impedance vectors of cancer patients and healthy subjects (i.e., the limit containing the magnitude and the phase angle of the mean vectors with 95 % probability). Two mean vectors, from two independent groups of subjects, were compared with the two-sample Hotelling’s *T* ^2^ test. Separate 95 % confidence limits indicate a statistically significant difference between mean vector positions on the *R*–*X*_c_ plane, i.e., in their *R*/*H*, *X*_c_/*H*, or both components or in their magnitude, phase angle or both (*P* < 0.05, which is equivalent to a significant Hotelling *T* ^2^ test) [21].

### Statistical methods

Our results are expressed as mean ± SD. The Shapiro–Wilk (S–W) test was used to assess the distribution conformity of examined parameters with a normal distribution; the Fisher (F) test was used to assess variance homogeneity. For group comparisons of metric data we used the Mann–Whitney *U* test. A *P* value <0.05 was considered statistically significant. The statistical analysis for this study was performed using the computer software STATISTICA v.8.0 (StatSoft, Poland). BIVA was done with BIVA software (version 2002).

## Results

As previously stated, many research studies refer to the great reproducibility of direct bioimpedance measurements (*R*, *X*, PA) at 50 kHz.

The characteristics of the H&NC and healthy subjects with average values of protocol variables are reported in Tables 1 and 2.

**Table 1:** Baseline characteristics of the H&NC patient and control group

Characteristic	Value (H&NC patients)	Value (control group)	*P*
Age at diagnosis (years)	56.75 ± 7.87	56.69 ± 13.11	NS
Subjective global assessment (SGA)	Well-nourished 37 (55)	Well-nourished 67 (100)	
	Moderately malnourished 24 (36)	Moderately malnourished 0 (0)	
	Severely malnourished 6 (9)	Severely malnourished 0 (0)	
	Unknown 0 (0)	Unknown 0 (0)	
BMI (kg/m^2^)	22.90 ± 4.37	23.22 ± 3.50	NS
Height (cm)	172.53 ± 6.26	171.69 ± 7.40	NS
Weight (kg)	68.11 ± 12.93	69.19 ± 11.34	NS
Serum albumin (g/dL)	4.03 ± 0.37	n/a	
Total protein (mg/dL)	7.14 ± 0.57	n/a	
Serum transferrin (mg/dL)	202.47 ± 39.63	n/a	
*R* at 50 kHz (Ω)	563.53 ± 91.08	526.57 ± 58.03	NS
*R*/*H* (Ω/m)	327.01 ± 54.13	307.43 ± 38.09	NS
*X*_c_ at 50 kHz (Ω)	48.32 ± 7.20	50.19 ± 8.91	NS
*X*_c_/*H* (Ω/m)	28.04 ± 4.26	29.29 ± 5.41	NS

*n* = 67; *x* ± SD; range in parentheses (all such values)

**Table 2:** Baseline characteristics of the H&NC patient and control group

Characteristic	*n*	Percent
Sex		
Male	67	100
Prior treatment history		
Newly diagnosed	67	100
Tumor stage at diagnosis		
Stage III	25	37
Stage IV	42	63

*n* = 67

There were no significant differences in mean values of age, weight, height and BMI between the two groups (H&NC and healthy subjects).

**Fig. 1:**
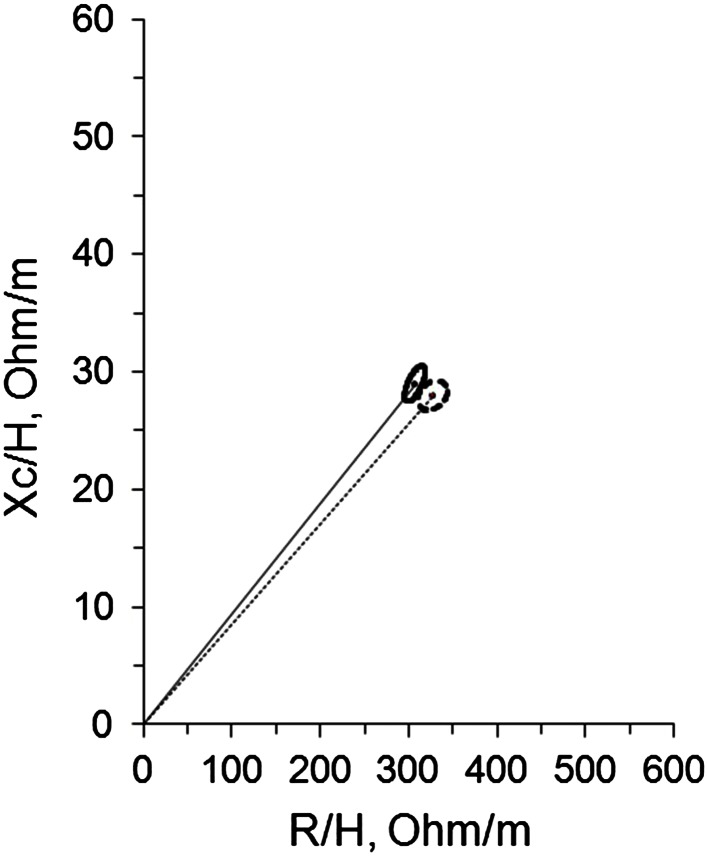
Mean vectors of 95 % confidence limits in H&NC patients (*dotted black line*) and healthy subjects (*solid black line*) (*T* ^2^ = 13.4, *P* < 0.0018)

As shown in Fig. 1, there was a significant displacement of the average impedance vector in cancer patients as compared with healthy controls, as indicated by separate 95 % confidence limits of mean vectors (*T* ^2^ = 13.4, *P* < 0.0018).

## Discussion

Malnutrition is known to be associated with adverse outcomes in cancer patients. In general, patients who have been and/or are being treated for H&NC have a compromised nutritional status [13]. BIA has been validated for the assessment of body composition and nutritional status in patients with cancer [14]. In this study, we observed a different vector distribution in H&NC group as compared with healthy subjects matched by sex, age and BMI. The vector displacement of patients with H&NC was characterized by a reduced *X*_c_ component and with increased *R* component (Fig. 1). The study by Toso et al. [10] reported that altered tissue properties might reflect previous complex systemic alternations induced by cancer. The observed impedance pattern indicated altered electrical properties of tissue, presumably of the body cell mass, because the *X*_c_ component of the impedance vector is determined mainly by dielectric properties of cell membranes of soft tissue [15–19]. In our group of patients, a pure disorder of soft tissue hydration cannot be ruled out, because the *R* component of the impedance vector was increased in comparison with the control group. Indeed, as documented in the literature, impedance vectors were longer and steeper in dehydration (e.g., after fluid removal by hemodialysis) [20–22]. In our population of H&NC patients, we observed that there was a smaller distribution of water between the extra- and intracellular compartments, and that there was a greater resistance of electric current due to the smaller distribution of water in these patients.

The hypothesis of altered tissue structure due to alterations induced by cancer is also consistent with findings by Kadar et al. [23].

The clinical usefulness of early detection of cancer metabolic activity independent of tumor mass would be determined by an increased precision of prognosis and the identification of subjects at risk for malnutrition and subsequent cancer cachexia, which can be useful in the tailoring of therapy. Our SGA results indicated that 55 % of this group was well nourished, 36 % moderately malnourished, and only 9 % severely malnourished. When one considers all available information from BIA, real malnutrition may be obscured by the subjectivity of SGA, and BIA may be a more sensitive measure of the nutritional status of cancer patients.

Our study was largely restricted to newly diagnosed patients. The results observed in our study provide valuable information on the nutritional status of the patient prior to surgery. Other methods of assessing nutritional status in this patient population, such as SGA, may not be sensitive enough to determine a deficiency. Previous studies, such as a study by De Luis et al. [24], were conducted on a population of Spanish ambulatory post-surgical male patients. However, there was not an evaluation of soft tissue hydration and mass through pattern analysis of vector plots as height, normalized resistance, and reactance measurements by BIVA. Their study did not indicate how long after the surgical procedure the BIA measurements were taken. The difference in the time period of performing BIA measurement is significant as post-operative patients may experience a rapid improvement in nutritional status.

Evaluating soft tissue hydration and mass through pattern analysis of vector plots as height, normalized resistance, and reactance measurements by bioelectric impedance vector analysis among pre-surgical H&NC patients can provide a quick, simple, and reproducible means to determine nutritional status. This quick assessment of the nutritional status of the patient can allow for early corrective intervention.

## Conclusion

Rapidly available, non-invasive, bioelectric impedance vector analysis (BIVA) may offer objective measures to improve clinical decision-making and predict outcomes. Monitoring vector displacement trajectory toward the reference target vector position may represent useful feedback in support therapy planning of individual patients before surgery in patients with H&NC patients to reduce post-operational complications due to malnutrition.

## Abbreviations

BIVA: Bioelectric impedance vector analysis
*R*: Resistance
*X*_c_: Reactance
PA: Phase angle
